# Characterizing heterogeneous forest structure in ponderosa pine forests via UAS-derived structure from motion

**DOI:** 10.1007/s10661-024-12703-1

**Published:** 2024-05-09

**Authors:** Laura Hanna, Wade T. Tinkham, Mike A. Battaglia, Jody C. Vogeler, Scott M. Ritter, Chad M. Hoffman

**Affiliations:** 1 Department of Forest and Rangeland Stewardship, Colorado State University, 1472 Campus Delivery, Fort Collins, CO 80523 USA; 2 United States Department of Agriculture Forest Service, Rocky Mountain Research Station, 240 W Prospect Rd, Fort Collins, CO 80526 USA; 3 Natural Resource Ecology Laboratory, Colorado State University, Fort Collins, CO 80523 USA; 4 Colorado Forest Restoration Institute, Colorado State University, 1472 Campus Delivery, Fort Collins, CO 80523 USA

**Keywords:** Drone, Groups, Variable density, Silviculture, Spatial pattern

## Abstract

Increasingly, dry conifer forest restoration has focused on reestablishing horizontal and vertical complexity and ecological functions associated with frequent, low-intensity fires that characterize these systems. However, most forest inventory approaches lack the resolution, extent, or spatial explicitness for describing tree-level spatial aggregation and openings that were characteristic of historical forests. Uncrewed aerial system (UAS) structure from motion (SfM) remote sensing has potential for creating spatially explicit forest inventory data. This study evaluates the accuracy of SfM-estimated tree, clump, and stand structural attributes across 11 ponderosa pine-dominated stands treated with four different silvicultural prescriptions. Specifically, UAS-estimated tree height and diameter-at-breast-height (DBH) and stand-level canopy cover, density, and metrics of individual trees, tree clumps, and canopy openings were compared to forest survey data. Overall, tree detection success was high in all stands (*F*-scores of 0.64 to 0.89), with average *F*-scores > 0.81 for all size classes except understory trees (< 5.0 m tall). We observed average height and DBH errors of 0.34 m and − 0.04 cm, respectively. The UAS stand density was overestimated by 53 trees ha^−1^ (27.9%) on average, with most errors associated with understory trees. Focusing on trees > 5.0 m tall, reduced error to an underestimation of 10 trees ha^−1^ (5.7%). Mean absolute errors of bole basal area, bole quadratic mean diameter, and canopy cover were 11.4%, 16.6%, and 13.8%, respectively. While no differences were found between stem-mapped and UAS-derived metrics of individual trees, clumps of trees, canopy openings, and inter-clump tree characteristics, the UAS method overestimated crown area in two of the five comparisons. Results indicate that in ponderosa pine forests, UAS can reliably describe large- and small-grained forest structures to effectively inform spatially explicit management objectives.

**Supplementary Information:**

The online version contains supplementary material available at 10.1007/s10661-024-12703-1.

## Introduction

Urban encroachment, shifting climates, and alterations to historical disturbance regimes in dry conifer ecosystems are accelerating changes to forest management approaches (Larson et al., 2012). Historically, forest management prioritized production and predictability, resulting in management practices designed to limit natural disturbance as well as structural and biological complexity (Fahey et al., 2018). This type of management largely resulted in expanses of dense, homogenous forests, making them more susceptible to severe biotic and abiotic disturbances (Allen et al., 2002). As disturbances have increased in frequency and severity, societal and managerial priorities have shifted toward restoring historical structural and biological diversity to promote ecosystem services, reduce the risk of large stand replacing fires, and promote resistance and resilience to a wide range of stressors (Franklin, 1993; Puettmann et al., 2010; Stephens et al., 2021). As a result, silvicultural prescriptions are increasingly focused on reestablishing the horizontal and vertical variability at both the tree-neighborhood and stand scale (Tinkham et al., 2017), while simultaneously calling for more frequent and widespread monitoring to facilitate adaptive management (Addington et al., 2018). However, the planning and design of effective heterogeneous prescriptions requires detailed quantification of the distribution and spatial arrangement of the forest overstory (Cannon et al., 2018).

Numerous methods of describing tree spatial arrangement have been explored for their ability to inform management decision-making. Through a comprehensive review of spatial patterns in forests that have frequent low-intensity fires of the Western United States, Larson and Churchill (2012) found that most (56 of 60 reviewed papers) studies employ global pattern analysis—or analysis strategies designed to describe spatial patterns through a singular stand-level metric. Given that horizontal heterogeneity in tree density, or groups and clusters, are a defining characteristic of these forests (Larson & Churchill, 2012), many studies employ spatial aggregation analyses such as Ripley’s K and Moran’s I. These strategies connect ecological processes like the facilitation and repulsion of species-specific regeneration to established tree patterns (Kuehne et al., 2015; Ziegler et al., 2017). Meanwhile, some studies focus on quantifying meadows and open space within stands as a function of the percent of stand area in open space (Matonis & Binkley, 2018), while others described the area of open space scaled by the Euclidean distance from trees (Churchill et al., 2013) or as a distribution of opening sizes (Cannon et al., 2018). Although these methods can provide some general idea of spatial patterns within stands, they often neglect open-grown trees and fail to describe vertical heterogeneity entirely. The lack of fine-scale characterization of the fuels complex associated with most stand-level approaches limits the ability to develop three-dimensional fuel representations at the stand scale required to inform silvicultural prescription development or the assessment of heterogeneous treatment effectiveness and longevity.

Analysis of local patterns is used to better describe stand structure and inform management actions. For instance, some studies have taken steps to identify and quantify local patterns of tree groups (Cannon et al., 2019; Tinkham et al., 2017). Other studies described tree arrangement as the distribution of clump sizes and characterizing tree size class variability within clumps (Larson & Churchill, 2012). These local pattern analyses often provide nuanced insights into stand structure and are easily integrated with silvicultural prescriptions or translated for treatment marking plans. One approach to describing and reintroducing mosaic patterns in dry conifer forests is the ICO (individuals, clumps, and openings) method initially developed by Larson and Churchill (2012). Such approaches require intensive stem map datasets to develop historical guidelines of clump size distributions for silviculture prescription development. However, because of high data collection costs, such comprehensive data are limited in both extent and temporal depth across dry conifer forests, limiting the implementation of such strategies.

Forest monitoring efforts now routinely augment field measurements with satellite sensor data (e.g., Landsat-9 or Sentinel-2 [Manfreda et al., 2018]). Even cutting-edge satellite sensors are currently unable to achieve the ultra-high spatial resolution (cm level) with multiple view angles that would be necessary to characterize individual tree size metrics other than crown area, especially in vertically heterogeneous forests (Freudenberg et al., 2022). Currently, satellite monitoring of forest structure lacks the range and versatility to meet the increasing demand for frequent ultra-high-resolution monitoring of forest structure (Manfreda et al., 2018). However, the increased focus on the spatial arrangement of tree groups in ecological restoration-based projects has led to a demand for data describing the current and dynamic arrangements of individual trees, clumps of trees, and canopy openings within heterogeneous forested landscapes (Camarretta et al., 2020; Castro et al., 2021).

In response, uncrewed aerial systems (UAS) have quickly risen as versatile alternative data collection platforms with the potential to bridge these spatial and temporal divides (Manfreda et al., 2018). Specifically, individual tree detection methods have been able to identify greater than 90% of trees in ponderosa pine (*Pinus ponderosa* var. *scopulorum* Dougl. Ex Laws.) forests, facilitating high-resolution tree-to-tree spatial arrangement analysis (Creasy et al., 2021). Additionally, the integration of diameter-at-breast-height (DBH) extraction and modeling strategies in UAS monitoring has made it possible to generate diameter distributions and stand basal area estimates within 10% of stem map observations (Swayze et al., 2021; Tinkham et al., 2022). Having near census-level forest inventories would enable managers to map explicit locations for tree retention and planned openings for use by marking crews or directly in the cabs of forest operations machinery (Keefe et al., 2022), eliminating potential subjectivity or second-hand interpretation of silvicultural prescriptions. Similarly, having tree-oriented continuous maps of forest structure holds the future of unlocking the use of next-generation fire behavior models in comparing treatment alternatives or planning prescribed fires (Moran et al., 2020; Pimont et al., 2016). However, the quality of individual tree remote sensing methods sometimes relies on offsetting tree detection errors, where trees that are missed (i.e., false negatives) are balanced against trees that are incorrectly added (i.e., false positives [Jeronimo et al., 2018]). Ideally, both errors are close to zero and equal to each other across size classes, resulting in a dependable representation of tree size distributions (Li et al., 2012). It is unknown how these tree-level errors will impact the data’s reliability in describing the local vertical and horizontal complexity.

This study evaluates the accuracy of UAS-estimated horizontal and vertical forest structural heterogeneity across a range of post-treatment forest structures in ponderosa pine-dominated forests. Specifically, we compare the spatial pattern of UAS single tree detection estimates against 11 stem-mapped 1-ha sites exhibiting a gradient of horizontal and vertical heterogeneity. Metrics are evaluated as tree-level DBH and height accuracy, distributions of clump sizes and vertical complexity, and stand-level density metrics.

## Methods

### Study site description

**Fig. 1:**
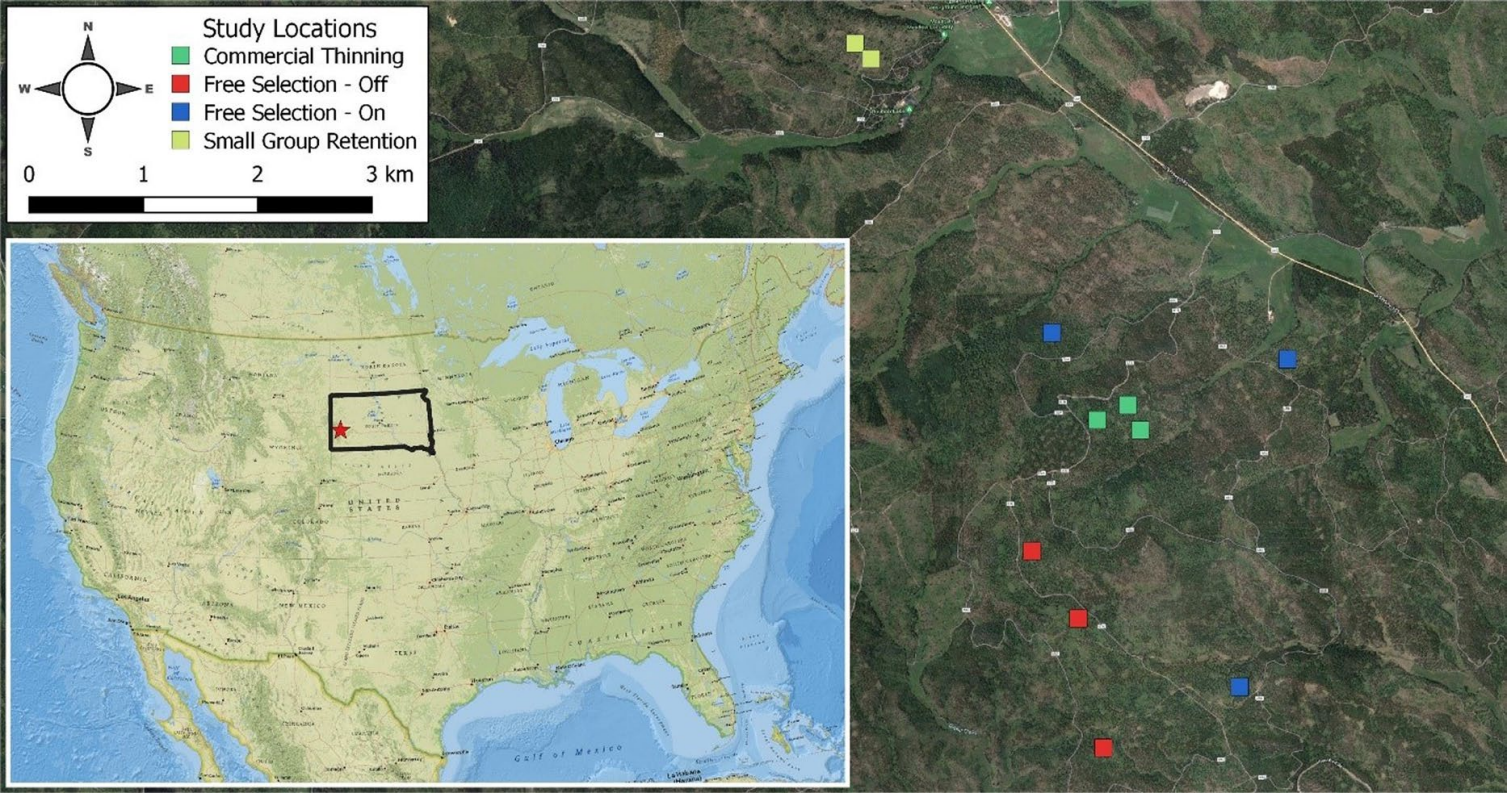
Study area showing the location of the eleven 1-ha study plots on the Black Hills National Forest in western South Dakota, United States. The red star in the inset map represents the general area within South Dakota that the study plots are located

In 2017, eleven 100 m × 100 m (1 ha) ponderosa pine-dominated plots were inventoried within the Black Hills Experimental Forest, a part of the Black Hills National Forest in western South Dakota, USA (Fig. 1). Study sites were selected to provide a wide range of local tree densities and height complexity for testing if these unique structures could be identified and characterized. The plots were randomly located within stand interiors (> 50 m from the stand edge) that had received one of four unique treatments designed to promote variation in forest structure horizontal and vertical complexity (Ritter et al., 2022). Ground plot mapping included observations of tree location, species, DBH, height, and crown width along the major and minor axis of each tree greater than 1.37 m tall. The crown area of each tree was estimated from its average crown width observation, and assuming the area of a circle, the crown areas were dissolved to eliminate crown overlap during stand and cluster analysis. Stem mapping was completed by establishing a 25 m × 25 m grid of survey locations in each stand with a Pentax PCS-515 (TI Asahi Co., Saitama, Japan) laser total station and then recording the northing and easting of each tree to a point in the survey grid with distance tapes. Further details of plot establishment can be found in Ritter et al. (2022).

**Fig. 2:**
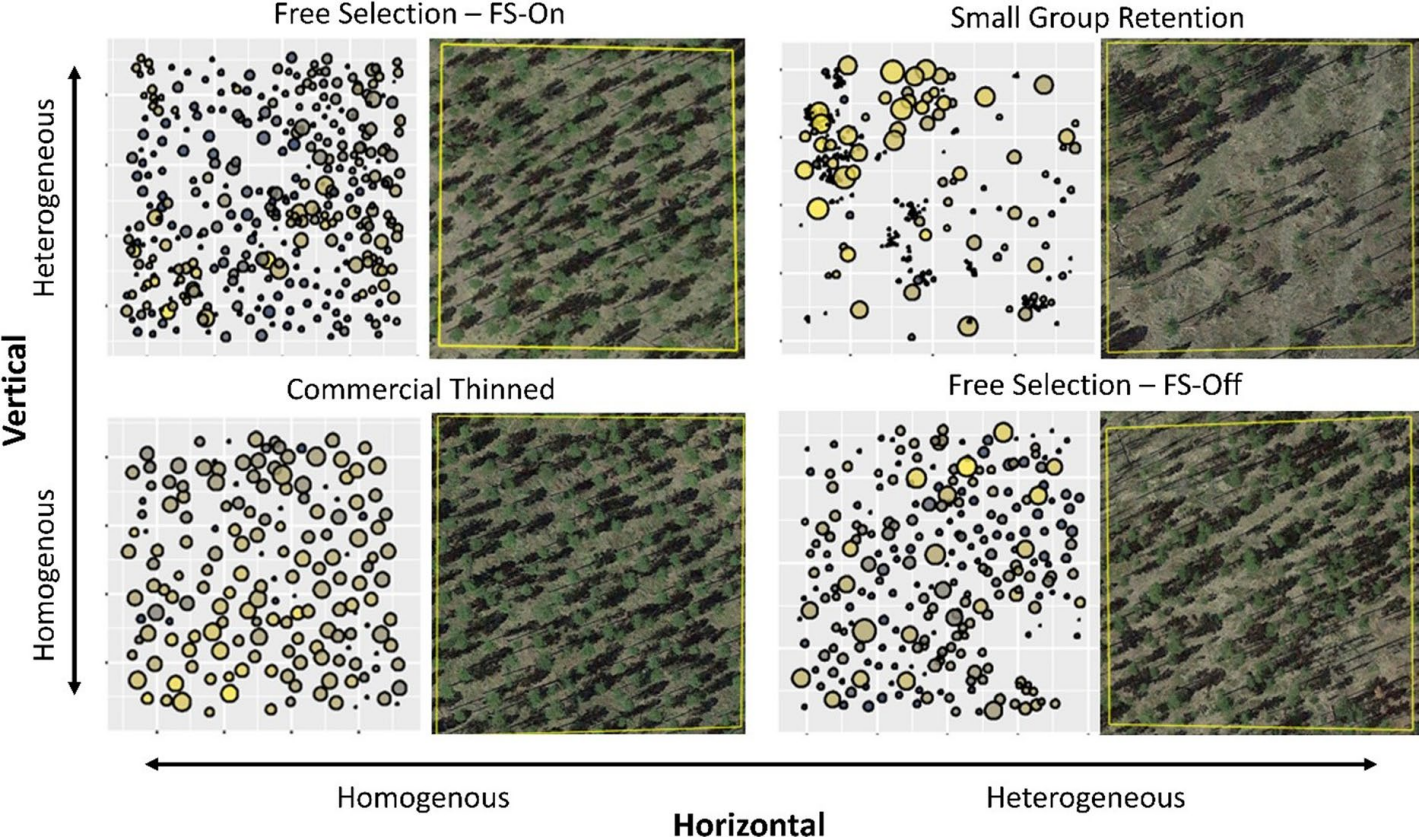
Stem map and aerial photo of representative plots for each of the treatment types. Sites are placed along relative scales from more homogeneous to more heterogeneous horizontal and vertical heterogeneity. Stem map trees are scaled according to their crown diameter

Study plots were in mechanically thinned forest treatments designed to capture a range of forest structure metrics (Fig. 2). The thinning treatments occurred between 2012 and 2014. These mechanical treatments consisted of small group retention (SGR), commercial-grade thinning (CT), and two free selection treatments. The SGR treatments called for the retention of ~ 4.6 m^2^ ha^−1^ of basal area (BA) with half of this in ~ 20 tree groups and half in scattered individuals. The retained groups emphasized large trees but also included trees of different sizes. In addition, pre-commercial sapling (0.1 to 12.4 cm DBH) and pole (12.4 to 22.6 cm DBH) sized trees were retained in large patches, resulting in both high vertical and horizontal heterogeneity (Fig. 2). The CT plots were thinned from below to a basal area of 9.2 to 13.8 m^2^ ha^−1^, and trees were spaced a minimum of ~ 4.9 m apart, resulting in both low vertical and horizontal heterogeneity (Fig. 2). Both free selection prescriptions (FS-On and FS-Off) called for thinning the commercial-sized trees (> 22.6 cm DBH) to 9.2 to 13.8 m^2^ ha^−1^ where ponderosa pine was favored for retention. These two free selection prescriptions used a crown vigor selection criterion (Graham & Jain, 2005; Hornibrook, 1939) to leave commercial-sized trees. However, they differed in their treatment of pre-commercial-sized trees (< 22.6 cm DBH) with the FS-On treatment ignoring the overstory when thinning pre-commercial stems to a fixed ~ 4.3 m spacing. In contrast, the FS-Off treatments thinned pre-commercial stems to an ~ 4.3 m spacing when considering both overstory and other pre-commercial stems. On FS-On sites, foresters were told to think of commercial stems as “ghosts” or to imagine that they were not there when considering tree spacing, whereas FS-Off sites included commercial stems in their tree spacing considerations. Overall, this resulted in greater vertical heterogeneity in the FS-On plots. Although each of the sites received one of these four treatments, these stands were selected to provide a gradient of tree size and group-level variation in structural complexity.

### UAS data collection and processing

**Fig. 3:**
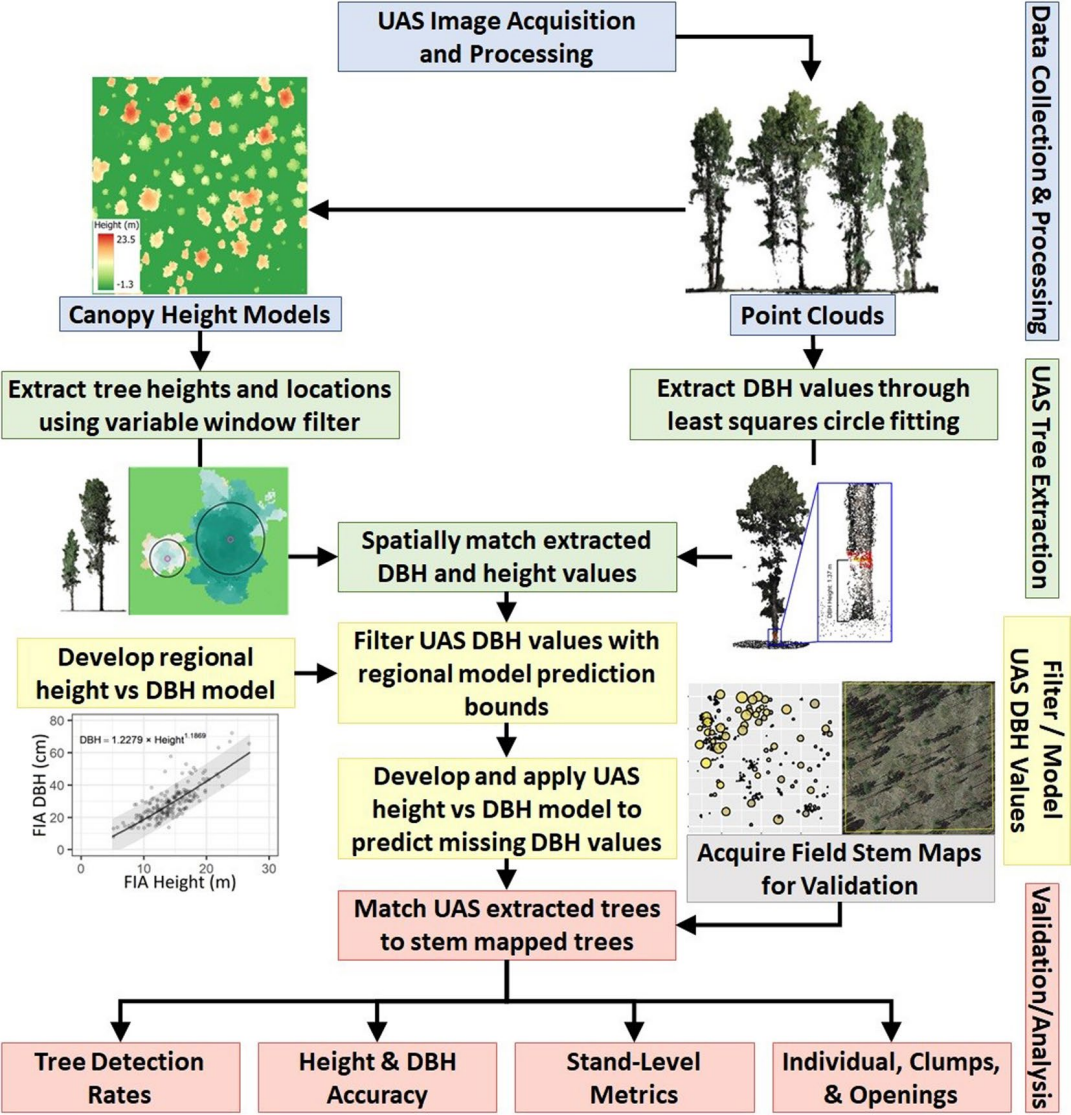
Workflow diagram showing the integration of UAS data collection, raw image processing through the structure from motion algorithm, extraction of individual tree height and DBH, filtering of DBH values with regional height to DBH model, prediction of missing DBH values from UAS modeled height to DBH relationship, and matching of UAS and stem-mapped trees for analysis of tree, stand, and clump level accuracy

In the summer of 2020, we planned and executed 11 flights over the 1-ha sites using a DJI Phantom 4 Pro to acquire very high-resolution (< 2.0 cm) imagery of each study site (Fig. 3). The Phantom 4 Pro was equipped with a 20-megapixel (5472 × 3648 pixels) metal oxide semiconductor (CMOS) red–green–blue sensor with a fixed 8.8-mm focal length. Altizure (version 4.6.8.193; Shenzhen, China) for Apple iOS was used to pre-program and conduct automated UAS crosshatch flight paths at an 80 m altitude, 90% forward and 85% side overlap at 4.0 m s^−1^ flight speed using a nadir (perpendicular to the ground) camera orientation. Flight boundaries were extended past the study boundaries to ensure constant image overlap throughout the 1-ha study site. Flights average approximately 3 min per hectare in the field. To improve georectification and image alignment, ground control points were established using an Emlid Reach-2 real-time kinetic GPS at approximately the center and four corners of each plot but shifted to ensure visibility in the UAS imagery when a tree canopy was present. The GPS base station never exceeded 2.5 km from the recorded points with PDOP ranging from 1.1 to 3.1, achieving an average reported horizontal root mean squared error (RMSE) of 0.41 m. The ground control points for two of the sites had large vertical errors (0.48 to 4.01 m RMSE) compared to the other sites (0.01 to 0.62 m RMSE) resulting in skewed height depth maps that overestimated tree heights by 5.0–7.0 m. These two sites were subsequently reprocessed without ground control points but still maintained a relative horizontal accuracy of less than 2.00 m RMSE when comparing UAS-detected tree locations with matched stem-mapped tree coordinates.

Images were processed using Agisoft Metashape Version 1.6.4 to generate structure from motion (SfM) point clouds following the methodology outlined by Tinkham and Swayze (2021). Study-specific processing parameters for Agisoft Metashape are reported in Supplemental Table A1. Point cloud generation through the structure from motion software required approximately 90 min for each study site, with larger acquisitions commonly averaging 30 to 40 min per hectare. The resulting SfM point clouds underwent processing in the lidR package (Roussel et al., 2020) for the R statistical program (R Core Team, 2022), including ground filtering, height normalization, and canopy height model (CHM) generation at a resolution of 0.10 m. Because the point cloud data density exceeded 2000 points m^−2^ for each study area, the point-to-raster function was used to generate the CHM from the height normalized point clouds. From the CHMs, individual trees were detected using a variable window function that reports tree location and height, following Creasy et al. (2021). The variable window function scales the search radius around each focal cell of the CHM using Eq. 1 to evaluate if the focal cell was the local maximum, where the focal cell needs to be the greatest value in the search radius to be retained as a tree location that is assigned the cells height value.

**Table 1:** Summary of stand estimates of tree density, size, and canopy cover for the field stem-mapped and UAS-extracted trees

Plot name	QMD (cm)		Trees ha^−1^		Trees ha^−1^ (> 5 m)		Basal area (m^2^ ha^−1^)		Canopy cover (%)	
	Stem map	UAS	Stem map	UAS	Stem map	UAS	Stem map	UAS	Stem map	UAS
SGR-1	16.2	17.2	307	195	84	78	6.4	4.5	12.8	10.4
SGR-2	11.2	15.5	658	362	151	146	6.5	6.9	12.6	12.9
FS-Off-1	23.7	18.2	251	387	206	221	11.1	10.1	17.8	21.7
FS-Off-2	24.0	19.4	254	420	168	196	11.5	12.5	22.8	21.5
FS-Off-3	22.4	20.9	263	323	225	239	10.3	11.0	15.7	19.2
FS-On-1	23.8	17.6	244	516	158	193	10.8	12.6	22.0	23.1
FS-On-2	20.3	17.1	348	540	288	284	11.3	12.3	20.3	28.7
FS-On-3	18.9	16.3	225	321	70	71	6.3	6.7	12.2	13.5
CT-1	28.4	26.0	171	196	158	163	10.9	10.4	24.1	19.0
CT-2	33.4	32.5	159	167	149	156	13.9	13.9	26.8	27.5
CT-3	28.2	22.0	189	222	179	202	11.8	8.4	24.7	24.6

1 $$Variable \;Window \;Radius = CHM \;Focal \;Cell \;Value \;\times 0.2$$

**Fig. 4:**
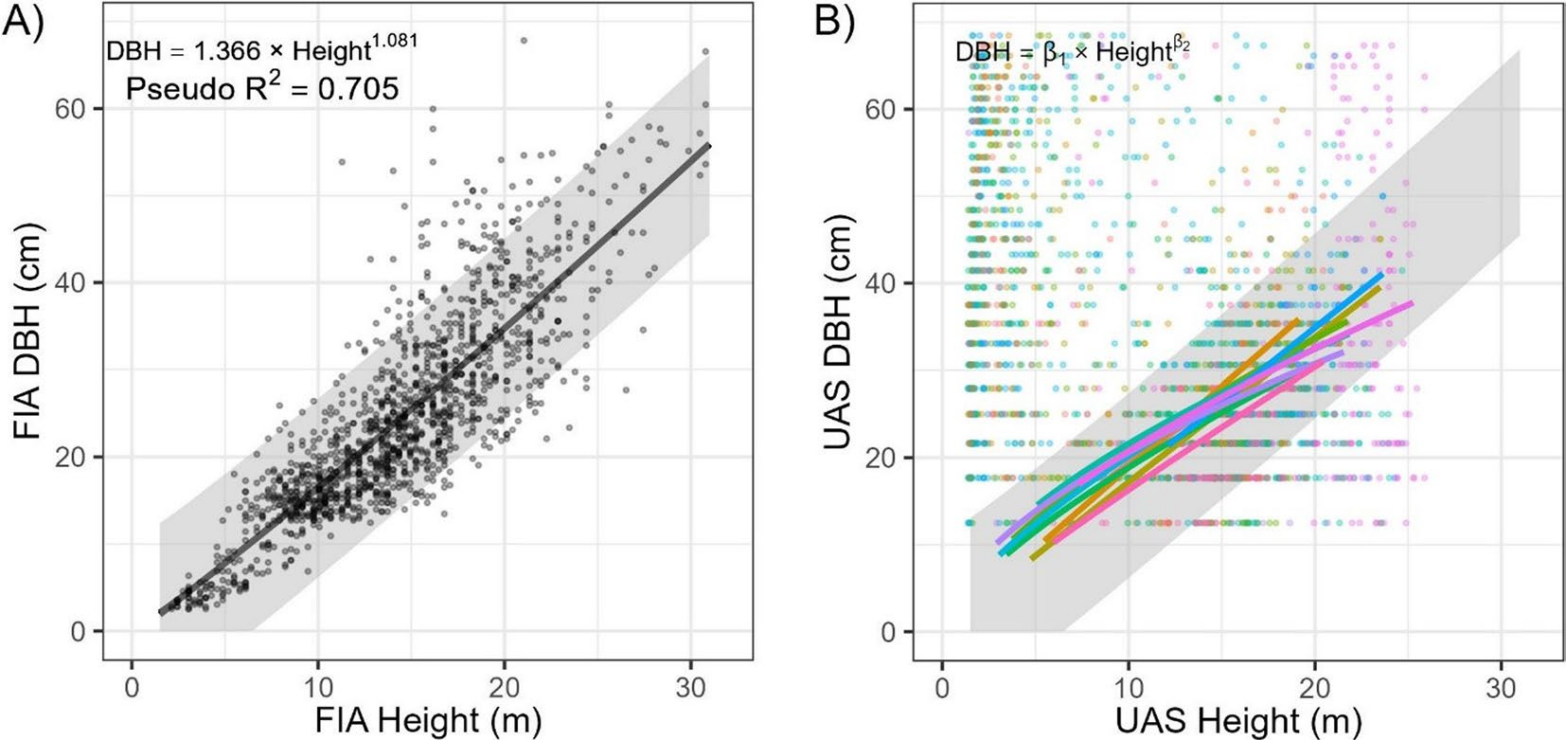
Modeled relationships of height predicting DBH for the **A** regional model using Forest Inventory and Analysis (FIA) data and **B** the 11 models fit to filtered UAS observations. The shaded area in both plots represents the regional FIA model’s 90% prediction bound used to filter UAS-extracted DBH values. The different colors in panel B represent the 11 stands being assessed, and only UAS height and DBH pairs that fell inside the prediction bound were used to fit a local height to DBH function and predict missing DBH values (2-column)

The DBH for each tree was modeled by adapting the workflow of Swayze et al. (2021). This approach uses the TreeLS package (Conto, 2019) to extract a slice of the height-normalized point cloud at 1.32 to 1.42 m, compresses the points to a flat plane, and then iteratively fits an ordinary least squares circle algorithm to each tree location to estimate DBH. However, this process can mistakenly fit circles across branches and is only expected to extract 10–20% of DBH values in ponderosa pine forests (Tinkham et al., 2022). To account for missing DBH values, regional United States Forest Service Forest Inventory and Analysis (FIA; Tinkham et al., 2018) data from the Black Hills National Forest were used to create a regional model of height predicting DBH (Fig. 4). The regional model was fit using a power function, achieving a residual standard error of 6.2 cm using the *nls* function in the stats package for the R statistical program. The successfully extracted UAS height and DBH pairs were filtered against the 90% prediction bounds of the regional model that was generated using the propagate package (Spiess, 2018) for the R statistical program. Only UAS DBH values falling inside the prediction bound were retained. Using the filtered UAS height and DBH pairs, site-specific power functions of height predicting DBH were created for each site to predict the missing DBH values for the remaining UAS-extracted heights (Fig. 4). These site-specific models had a mean pseudo-*R*^2^ of 0.51 (SE = 0.04) and mean residual standard error of 1.20 cm (SE = 0.01 cm), with a mean β_1_ of 4.29 (SE = 0.40) and mean β_2_ of 0.68 (SE = 0.04). Finally, individual tree crown areas were estimated from the UAS CHM using the marker-controlled watershed method of the ForestTools package (Plowright & Roussel, 2021), providing a UAS dataset containing the location, DBH, height, and crown area of extracted trees. The processing from raw point cloud to UAS-derived inventory with location, height, and DBH for each tree requires approximately 80 min per hectare.

### Tree matching and error assessment

Extracted and modeled UAS tree observations were spatially matched with individual stem-mapped trees following Silva et al. (2016) to provide an evaluation of true positive, false positive, and false negative rates and to compare tree and stand-level structural attributes. These spatially aligned datasets were further used to evaluate the ability of the UAS inventory to describe horizontal and vertical structural complexity by comparing the distribution of UAS-detected tree clusters and openings against distributions derived from the stem-mapped trees.

Matching of UAS trees with field stem map trees was conducted by selecting a target UAS tree and identifying all candidate stem map trees within 4.0 m. If one or more candidate stem map tree was found to have less than a 2.0-m height error, the candidate with the smallest error was assigned as a true positive match and removed from the process. If a match could not be found for the target tree, the target was considered a false positive. The process considered each UAS tree iteratively until all UAS trees were classified as true positive or false positive. All remaining stem map trees that could not be matched were considered false negatives. Based on the calculated true positive, false positive, and false negative rates, *F*-score was calculated as an overall metric of tree extraction success using Eq. 2. Our *F*-score, true positive, false positive, and false negative rates were then summarized across understory (< 5.0 m tall), intermediate (5.0–15.0 m), and overstory (> 15.0 m) dominance classes.

2 $$F\text{-}score =2\times \frac{\left(\frac{True \;Positive}{True \;Positive \;+ \;False \;Negative} \times \frac{True \;Positive}{True \;Positive \;+ \;False \;Positive}\right)}{\left(\frac{True \;Positive}{True \;Positive \;+ \;False \;Negative} \;+ \;\frac{True \;Positive}{True \;Positive \;+ \;False \;Positive}\right)}$$

Using the matched field stem map and UAS-extracted trees, the mean error (ME) and root-mean-squared error (RMSE) of tree height and DBH were calculated for each study site. To evaluate how tree height and DBH errors vary across tree size, observations were summarized across 5-m tree height size classes. Finally, to evaluate the efficacy of UAS measurements for stand-wide data collection and analysis, we compared estimates of stand basal area and trees per hectare (TPH), quadratic mean diameter (QMD), and percent canopy cover between the stem-mapped and UAS-extracted trees for each site. Remotely sensed canopy cover was defined as the proportion of CHM pixels identified as crown within each site, while the elliptical area of stem-mapped tree crowns was spatially intersected to eliminate canopy overlap before determining canopy cover.

To evaluate the ability of UAS-extracted trees to characterize horizontal and vertical heterogeneity tree arrangement, clusters of trees were identified within the stem-mapped and UAS trees. A cluster of trees was defined as two or more trees with the potential for interlocking crowns. Overstory trees on the stem maps generally had a crown radius of ~ 3.0 m, so stems within 6 m of one another were considered to have the potential for developing interlocking crowns. Density-based spatial clustering of applications with noise (DBSCAN) from the fpr package (Hahsler et al., 2019) in R was used to assign trees to unique clusters, including individual trees that were assigned by themselves if they did not have the potential to develop interlocking crowns (> 6 m from another tree).

To analyze the effect of tree aggregation on tree attributes, the identified trees and clusters were designated as an “individual” or as a cluster consisting of 2–4, 5–9, 10–15, and > 15 trees. We then calculated the number of clusters, the percent of stand basal area, the height coefficient of variation, and the canopy area within the cluster size classes for each site. Differences in the mean UAS and stem-mapped datasets of these metrics were compared through a series of one-way analyses of variance (ANOVA). All analyses of variance used a Bonferroni correction to determine adjusted *p*-values (Eq. 3) for identifying significant differences (*α* ≤ 0.05). The Bonferroni correction was included to account for the increased type 1 error rate associated with running the ANOVA across multiple levels. Comparisons of the number of clusters and canopy area were summarized at the study site level providing *n* = 11 for the five comparisons across clump sizes. Comparisons of the cluster basal area and height coefficient of variation were done using the individual clusters, providing a minimum of *n* = 35 and five clump size comparisons for basal area and four clump size comparisons for height coefficient of variation.

3 $$Bonf\text{-}adj \;p\text{-}value =\# \;of \;comparisons\times \;p\text{-}value$$

Finally, to assess the efficacy of UAS for describing openings within each plot, distributions of inter-tree distances to every location in a 1.0-m grid were determined for each dataset. Distance distributions were used to calculate the proportion of the total plot area within 3-m intervals of distance away from a tree. The total plot area detected within each distance interval was compared between the UAS and stem map datasets (*n* = 11) using a series of one-way ANOVAs with a Bonferroni correction for the five comparisons (0–3, 3–6, 6–9, 9–12, and 12–15 m).

## Results

### Tree and stand summarization

**Fig. 5:**
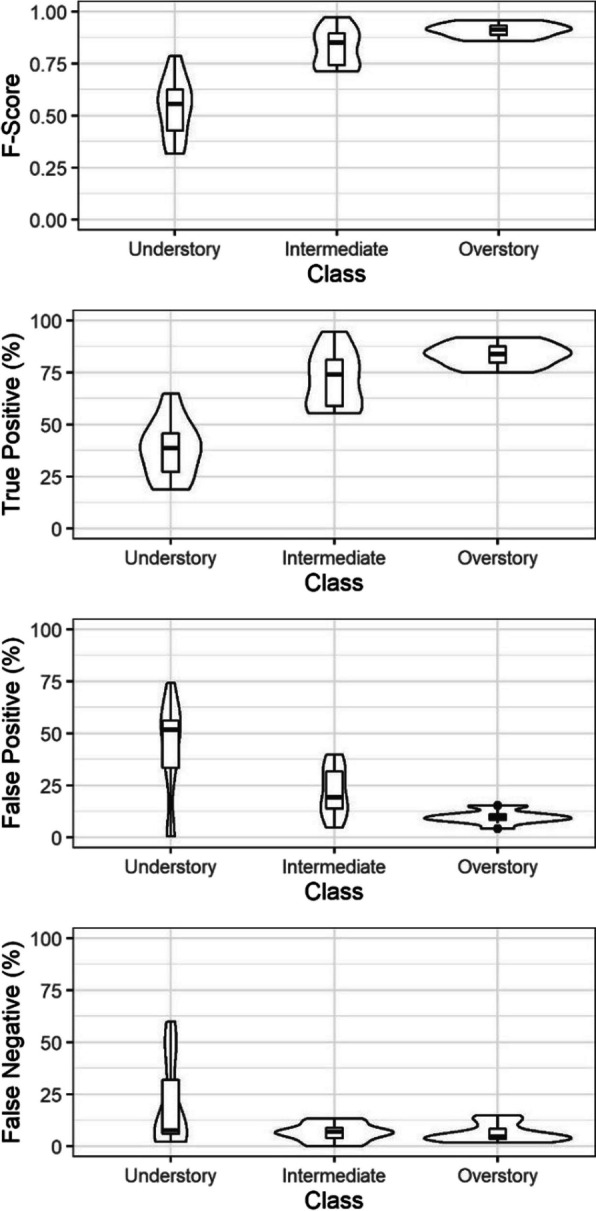
Summary of UAS tree extraction across understory (< 5.0 m), intermediate (5.0–15.0 m), and overstory (> 15.0 m) tree dominance classes. Each violin and nested boxplot represents the 11 observations from the different study sites, where each violin represents the complete distribution, and the nested boxplot shows the median and interquartile range

UAS tree detection resulted in *F*-scores from 0.64 to 0.89 across the 11 sites, with the *F*-score decreasing from the tallest to shortest height dominance classes (Fig. 5). For the intermediate and overstory classes, the average *F*-score exceeded 0.80, and the maximum exceeded 0.95. Within the understory class, *F*-score ranged from 0.31 to 0.80. Similar trends occurred for true positive rates, with performance maximizing in the tallest class and decreasing to the shortest class (Fig. 5). At the stand level, false positive and false negative detection rates were partially balanced and resulted in average values of 29.5 and 16.8%, respectively (Fig. 5). However, the smallest size class had greater false positive rates with an average of 61.4%.

**Fig. 6:**
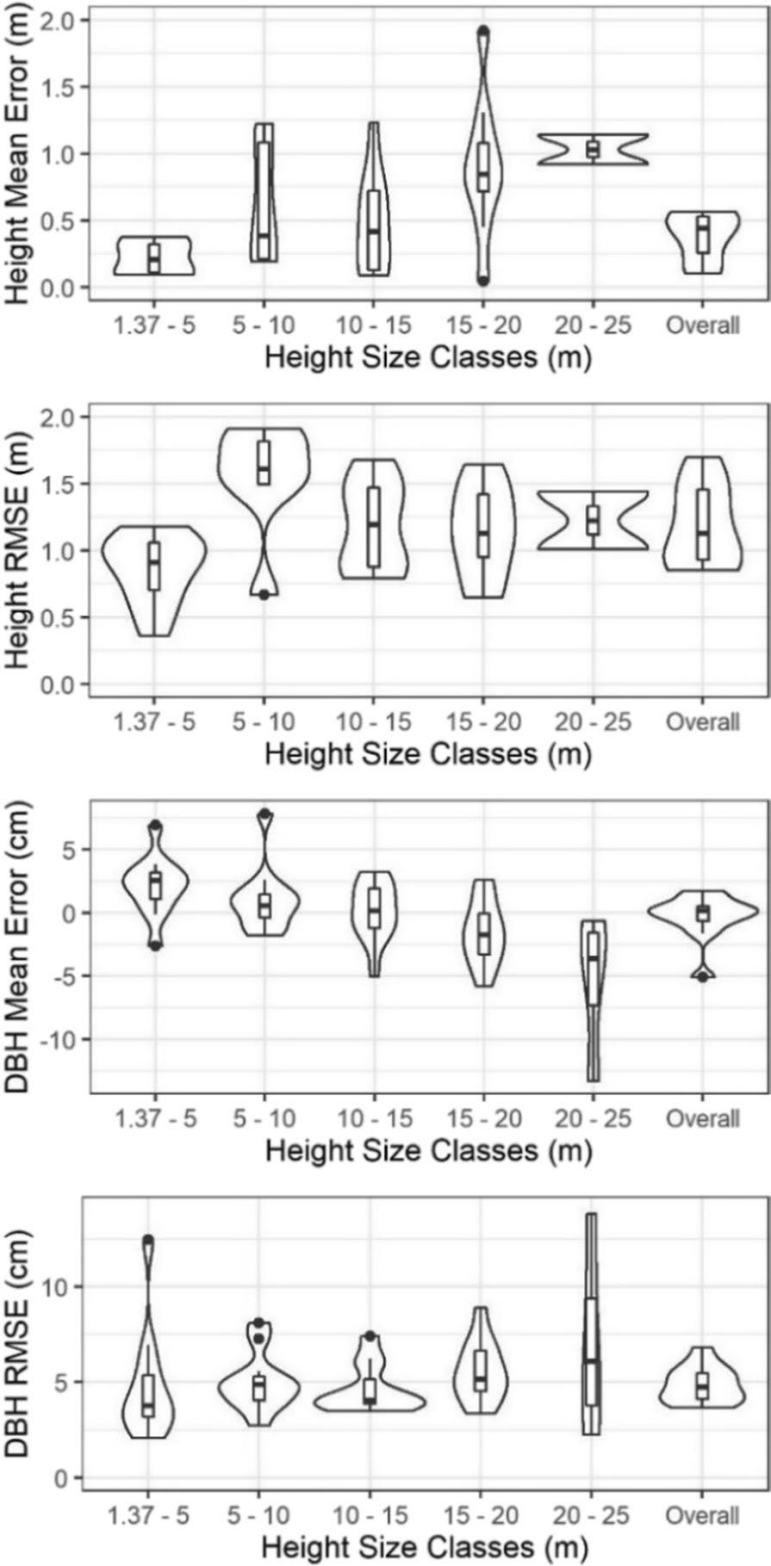
Summary of UAS-extracted tree height and DBH. Each violin and nested boxplot represents the 11 observations from the different study sites, where each violin represents the complete distribution, and the boxplot shows the median and interquartile range

Stand-level UAS tree height estimates tended to be slightly taller than the stem-mapped values with an average mean error of 0.36 m and average RMSE of 1.32 m (Fig. 6). Though mean error tended to increase as tree height increased, the RMSE was similar across the tree size classes. The tree-level DBH mean error across the stand averaged ~ 0.0 cm with the UAS DBHs tending to be overestimated for the smaller tree size classes and underestimated for the largest size classes (Fig. 6). Prediction of the missing DBH values using the UAS height to DBH model resulted in an average RMSE of 4.8 cm. When summarized across the 11 sites, the UAS-estimated QMD was on average underestimated by 2.5 cm (Table 1). Only the SGR treatments resulted in overestimated QMD values.

The 11 sites had an average TPH of 332, with values ranging from 159 to 658 TPH. The UAS observations overestimated TPH compared to the stem maps by an average of 53 TPH (27.9%), but this varied across the sites and treatments (Table 1). The largest overestimations in TPH were in the FS-On and FS-Off treatments at 187 and 121 TPH on average, respectively. Conversely, the small-group retention treatments underestimated TPH on average by 204, while the commercial thinning treatments were on average overestimated by 22 TPH. The free selection sites where TPH was overestimated had the greatest false positive rates in the understory size class (< 5.0 m tall), while the underestimated SGR sites had the largest false negative rates in the same size classes (Fig. 5). When only evaluating the TPH of trees greater than 5.0 m in height (Table 1), the UAS errors improved to an average underestimation of 10 TPH (5.7%) and RMSE of 17 TPH (10.1%).

Stand basal area estimates across all trees were similar between the UAS and stem maps with a mean underprediction error of 0.1 m^2^ ha^−1^ or − 1.7% (Table 1) and a mean absolute error of 1.1 m^2^ ha^−1^ or 11.4%. Although two stands showed greater underprediction errors at about − 29% and another overpredicted by ~ 16% for stand basal area, these errors were inconsistent with the errors seen for other sites with the same treatment type. The other nine sites all had errors of < 10%. Stand-level canopy cover estimates from the UAS ranged between underestimating by 5.1% and overestimating by 8.4% with a mean absolute error of 2.6% (Table 1).

### Characterization of horizontal and vertical heterogeneity

**Fig. 7:**
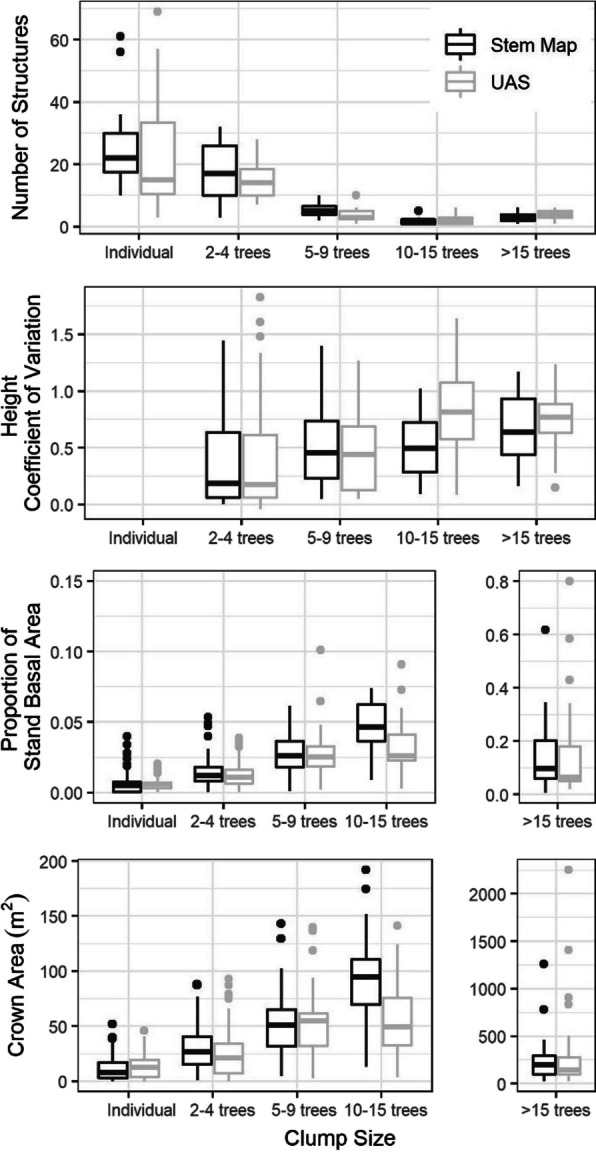
Evaluation of stem-mapped and UAS-extracted tree clusters, presented from top to bottom as boxplots showing the number of unique cluster structures, coefficient of variation for height within the clusters, the proportion of stand basal area within the clusters, and the crown area within clusters. The boxplots show the median and interquartile range

When evaluating the horizontal arrangement of trees, no significant differences were found between the stem maps and UAS data for the number of clumps within each of the clump size classes (Table 2, Fig. 7). Additionally, although close in one clump size class (*p* = 0.080), there were no significant differences in the coefficient of variation of tree heights within the different clump sizes (Table 2, Fig. 7). In describing the proportion of stand basal area within each clump size class, there were no significant differences between the UAS and stem map datasets (Table 2, Fig. 7). However, comparing the crown area in each clump size class between the datasets showed that the UAS method significantly underestimated the crown area in the 2–4 trees (*p* = 0.030) and 10–15 trees clump sizes (*p* = 0.010; Table 2). Across all intra-clump metrics, the 10–15 tree clump size consistently provided the largest differences between the datasets, although only significant for crown area (Table 2). Visual inspection of the data demonstrated that the observed differences (both significant and non-significant) can mostly be attributed to the 3-year delay between stem mapping and UAS data collection. This delay allowed a cohort of small trees to grow past 1.37 m tall, changing the number of trees in a clump in a way that either shifted them between clump classes or increased the clump’s height coefficient of variation.

**Table 2:** Summary of one-way ANOVAs comparing the distribution from the UAS and stem map datasets for the number of clumps by size, the proportion of stand basal area within clumps, height coefficient of variation within clumps, and the proportion of stand area at different distances from the nearest tree. Analysis used a Bonferroni correction to determine adjusted *p*-values, with significant differences (*α* ≤ 0.05) indicated in bold. Values are reported as mean (standard deviation) in the stem map and UAS columns

Metric and class		Data source		*F*	Bonf-adj *p*-value
	*n*	*Stem map*	*UAS*		
*Number of clumps*					
Individual trees	11	28 (17)	25 (21)	0.126	1.000
2–4 trees	11	18 (10)	15 (7)	0.435	1.000
5–9 trees	11	5 (2)	4 (3)	1.248	1.000
10–15 trees	11	2 (1)	3 (2)	0.301	1.000
> 15 trees	11	3 (2)	4 (2)	0.402	1.000
Height coefficient of variation (%)					
2–4 trees	330	38.3 (18.1)	34.3 (17.4)	0.077	1.000
5–9 trees	93	48.3 (24.3)	52.0 (26.9)	0.735	1.000
10–15 trees	26	50.4 (28.5)	88.3 (40.1)	5.832	0.084
> 15 trees	56	63.2 (26.3)	73.4 (22.7)	0.896	1.000
Proportion of stand basal area (%)					
Individual	597	0.5 (0.2)	0.5 (0.1)	0.215	1.000
2–4 trees	330	1.3 (0.3)	1.1 (0.3)	0.111	0.555
5–9 trees	93	2.8 (0.7)	2.4 (1.1)	0.779	1.000
10–15 trees	26	4.4 (2.0)	3.2 (0.7)	0.053	0.265
> 15 trees	56	15.5 (7.2)	14.3 (6.9)	0.941	1.000
Crown area (m^2^)					
Individual	11	11.2 (10.0)	13.2 (10.2)	5.721	0.085
2–4 trees	11	29.0 (18.9)	23.5 (18.5)	7.755	**0.030**
5–9 trees	11	52.4 (29.1)	51.5 (30.9)	0.025	1.000
10–15 trees	11	99.9 (46.1)	57.2 (35.4)	10.955	**0.010**
> 15 trees	11	246.5 (250.9)	290.6 (426.8)	0.250	1.000
Proportion of stand at distance to a tree (%)					
< 3 m	11	50.3 (9.7)	53.5 (12.3)	0.471	1.000
3–6 m	11	40.9 (6.4)	38.3 (8.3)	0.679	1.000
6–9 m	11	7.3 (5.6)	6.8 (6.5)	0.029	1.000
9–12 m	11	1.3 (1.6)	1.3 (2.1)	0.000	1.000
> 12 m	11	0.2 (0.4)	0.2 (0.5)	0.037	1.000

**Fig. 8:**
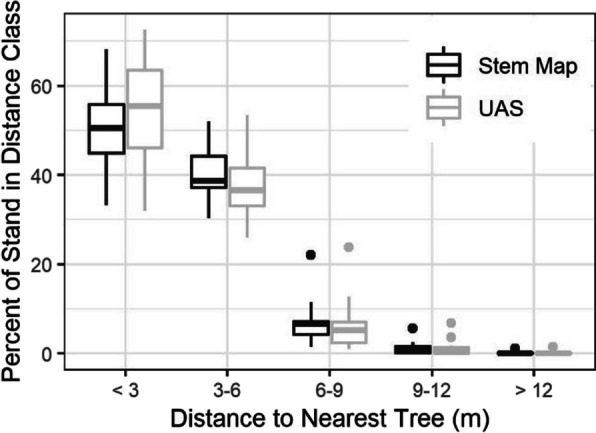
Boxplots showing the distribution of distance to the nearest tree within the stem-mapped and UAS-extracted tree datasets. The boxplots were developed from the 11 study sites and show the median and interquartile range of distance to the nearest tree within consecutive 3.0-m wide bands

Assessment of stand openings showed no significant differences in the proportion of stand area at different distance intervals from the nearest tree between the stem map and UAS datasets (Fig. 8, Table 2). The largest shift occurred in the < 3.0 m and 3.0–6.0 m distance intervals of ~ 6% over and underestimation, respectively.

## Discussion

Results demonstrate that UAS-based observations of ponderosa pine forest structural heterogeneity can be consistently achieved for a broad range of forest structures. Specifically, we evaluated tree detection rates and extracted height and DBH errors across tree size classes, finding that height and DBH estimates were well captured, with mean errors of 0.36 m and ~ 0.0 cm, respectively. Then we compared UAS-derived stand-level TPH, basal area, QMD, and canopy cover estimates against stem-mapped estimates. Tree detection was within 6% of tree ha^−1^ for stems > 5.0 m tall, with larger errors for sites with a greater density of shorter trees. These accuracies translated to mean absolute errors of 11.4% for stand basal area and 16% for QMD. Finally, we demonstrated that UAS methods can successfully characterize the distributions of individuals, clumps, and openings as well as the inter-clump characteristics of percent of stand basal area and height CV associated with clump size classes.

### Tree detection performance

Overall, the UAS-detected trees strongly agreed with the stem-mapped trees across the 11 sites, producing *F*-scores ranging from 0.64 to 0.89. Our tree detection success is comparable to findings from recent studies extracting individual trees from point clouds in pine-dominated forests that have produced *F*-scores ranging from 0.71 to 0.94 (Creasy et al., 2021; Mohan et al., 2017; Silva et al., 2016). This study differed from past studies, in that it was designed to assess if the wide range of forest structures and local aggregation levels produced through treatments in ponderosa pine forests could be characterized by UAS. We assessed the accuracy of data collection for trees of all size classes within both even and uneven-aged stand structures across densities ranging from 159 to 658 TPH. In our study, only trees detected within 2.0 m of height and 4.0 m of horizontal distance to a stem-mapped tree were considered true positive matches. Mohan et al. (2017) achieved an overall *F*-score of 0.86 in an open-canopy lodgepole pine-dominated (*Pinus contorta*) stand through a visual assessment of UAS-detected trees versus UAS-derived orthomosaics, point clouds, and crown height models. This approach may have had a higher likelihood of introducing human error as only trees that were visible in the orthomosaic could be assessed. Creasy et al. (2021) and Silva et al. (2016) both took similar approaches to this study’s tree matching method and achieved *F*-scores of 0.69–0.79 and 0.83, respectively. The Creasy et al. (2021) study occurred in untreated uneven-aged ponderosa pine stands and included all trees > 1.37 m tall, while the Silva et al. (2016) study occurred in longleaf pine (*Pinus palustris*) stands and only included trees > 6.0 m tall and at a lower stand density. The ability for this study and Creasy et al. (2021) to achieve similar results to other studies only including larger trees is attributed to the use of a fine resolution (0.10 m) CHM that facilitated the use of the variable window function during tree detection.

### UAS-extracted height and DBH

Extracted tree heights saw similar success as other UAS studies, with an overall RMSE of 1.32 m for extracting tree heights. This precision is in line with results from recent studies producing RMSE ranging from 1.30 to 3.94 m in dry eucalypt, ponderosa pine, and northern European mixed-conifer forests (Belmonte et al., 2020; Panagiotidis et al., 2017; Wallace et al., 2016). However, our study had an overestimation bias of 0.36 m which is double the 0.15 m seen by Swayze et al. (2021) and 0.13 m by Krause et al. (2019) in ponderosa pine and Scots pine (*Pinus sylvestris* L.) stands, respectively. Both of these studies acquired their UAS data within 1 year of their field data, whereas our increased overestimation bias is attributed to the 3 years between field stem mapping and UAS acquisitions, with a recent study in adjacent stands finding that ponderosa pine regeneration height growth averages 0.60 m every 3 years (Tinkham et al., 2021). Along with this, during plot stem mapping, it was noted that some sites had many trees between 1.00 and 1.30 m tall, but shorter than the 1.37 m threshold for inventorying at the time. As a result, many trees that were too short to be inventoried initially could have grown to be over 1.37 m by the time the UAS inventory occurred 3 years later. Additionally, any growth over these 3 years would lead to the consistent positive bias across all size classes that we observed.

The implemented flight, processing, extraction, and filtering procedure for DBH extraction correctly identified 26.3% of all tree DBHs on average across the 11 sites that corresponded with the regional height to DBH model’s prediction bounds. This extraction rate is more than three times greater than that achieved by Swayze et al. (2021) in untreated and overstocked ponderosa pine forests. However, looking at Fig. 4b, the importance of DBH filtering with the regional prediction bound is highlighted by the many erroneous DBH values that were eliminated in alignment with short tree heights that visual inspections showed to be patches of regeneration. After predicting these erroneous and missing DBH values, our 4.8 cm RMSE is on-par with past UAS studies conducted in coniferous systems with RMSE ranging from 3.5 to 4.2 cm (Brede et al., 2017; Dalla Corte et al., 2020). Similar trials have been conducted in broadleaf systems to varying results with RMSE ranging from 15.0 to 42.0 cm depending on scan angle and leaf presence (Neuville et al., 2021). All of the referenced studies, except Swayze et al. (2021), employed airborne LiDAR sensors, indicating that aerially acquired SfM point clouds are a comparably effective approach for DBH estimation. In dissecting our DBH errors, the shift toward underpredicting diameters for the tallest trees (Fig. 6) is attributed to many of these trees representing mature ponderosa pine with characteristic flat-topped crowns that had stopped accumulating height while still adding stem diameter. Similar error structures were observed by Tinkham et al. (2022) who suggested that moving beyond the power function model of height predicting DBH by including covariates of local stem density or crown structural attributes might improve the DBH prediction accuracy. Unfortunately, the current study only acquired post-treatment UAS imagery and thus could not explore how including local density metrics might improve DBH modeling, but future studies could track this through pre- and post-treatment monitoring to potentially enhance DBH modeling.

### UAS-estimated stand metrics

Overall, UAS monitoring successfully described stand-wide metrics of forest density, tree size, and cover (Table 1). However, we observed fluctuating error levels for TPH across treatments from 5 to 112% absolute error. The UAS method overestimated TPH in every plot except the SGR treatments, where UAS underestimated TPH by a wide margin. After excluding all trees < 5 m in height from TPH estimates, UAS absolute errors were reduced to < 8%—indicating that small stems were the primary source of error. The resulting under- and overestimation suggests two different error sources. Underestimation of TPH in the SGR treatments is likely due to limitations in identifying individual small stems in CHMs due to issues with separating the interlocking crowns in the high-density groups produced in this treatment (Creasy et al., 2021). Only area-based point cloud modeling techniques (Swayze et al., 2022) may be possible of characterizing these high regeneration density areas. The overestimation of TPH in the other treatments is attributed to the ingrowth of small trees past the 1.37 m tall threshold during the 3 years between stem mapping and UAS acquisitions.

Despite errors concerning understory TPH estimates, it seemed to only negligibly impact our ability to describe other stand-level metrics. Our mean absolute error in basal area of 11.4% is in line with results reported in pre-treatment ponderosa pine forests where stand-level basal area estimates were within 4.1 to 24.7% of field observed values (Swayze et al., 2021) and outperforms results from complex pre-treatment mixed hardwood and conifer forests where basal area was overestimated by 14.6 to 42.1% (Fraser & Congalton, 2021). Additionally, UAS estimates of QMD varied from 0.9 to 6.2 cm absolute error. Stand QMD was overestimated in the SGR treatments where some smaller trees were missed by the UAS and QMD was underestimated in treatments with larger proportions of mature trees where DBH values tended to be underestimated.

Stand-level canopy cover estimates were in line with field observations providing a mean absolute error of 2.6%. This follows other UAS-based approaches that have found LiDAR estimates of canopy cover to fall within 5% of field observations (Ahmed et al., 2015). Overall, remote sensing strategies have consistently been shown to effectively assess crown cover, as across different methods, studies have found strong relationships to field observations with *r*^2^ values ranging from 0.78 to 0.91 (Dickinson et al., 2016; Gülci, 2019; Tang et al., 2019). The relatively larger error in the UAS-estimated canopy area of the 10–15 tree class is at least partially attributed to ingrowth shifting groups between cluster classes. This shifted some groups into the 10–15 tree class with small canopy area values and shifted some groups into the > 15 tree class, ultimately causing the distribution of canopy area values in the 10–15 tree class to shift toward smaller values. Similarly, while it was not significant at the *α* = 0.05 level, it can be seen in Table 2 that the UAS derived a greater amount of height variation in the 10–15 tree class. Inspection of these group structures showed a number of trees that likely grew past the 1.37 m height threshold during the 3 years between stem mapping and UAS acquisition. Because these trees were excluded at the time of the field inventory but included by the UAS, they are attributed with increasing the group variation.

### Implications for management

This study demonstrated a UAS method for extracting spatially explicit tree lists across a range of treatments designed to create variation in horizontal and vertical heterogeneity. While this study was not able to assess the method’s performance in both pre- and post-treatment conditions, previous work using similar UAS strategies has demonstrated comparable, but slightly lower pre-treatment tree and stand structural attribute accuracies (Creasy et al., 2021; Swayze et al., 2021). Although not representing a complete census, such tree lists represent a valuable resource for land managers in planning, implementing, and evaluating spatially explicit silvicultural prescriptions (Addington et al., 2018). This level of data would enable managers to map explicit locations for tree retention and planned openings for use by marking crews or directly in the cabs of forest operations machinery (Keefe et al., 2022). The benefits of this type of spatially explicit data for treatment implementation are also highlighted in the individuals, clumps, and openings implementation guide for dry conifer restoration (Churchill et al., 2016) and the UAS approach used here provides a more flexible strategy for project level monitoring than landscape level airborne LiDAR acquisitions. This flexibility can facilitate greater use of pre- and post-treatment data collection to evaluate treatment implementation and improve future prescriptions. While not successfully extracting all trees from the UAS data, the presented methods captured the relative local trends and stand-level metrics that are necessary for informing a broad range of thinning and restoration (Almeida et al., 2019) actions in low to moderate canopy cover (e.g., < 60%) pine-dominated or mixed-conifer systems. The achieved precisions for basal area and trees per hectare fall well inside the common US public land forest inventory design standard of ± 20% allowable error at a 95% confidence level (USDA Forest Service, 2015).

While UAS understory stem density precision is flawed due to interlocking crowns in small tree groups, the methods tested in this study accurately reflect relative understory densities across the stand. Characterization of relative understory density across a stand has been proposed as sufficient to guide thinning objectives that target understory trees (Allen et al., 2002), especially as traditional field plot sampling only provides estimates of average stem density in a stand but not the stem locations.

The level of inventory information available from the approach we implemented can act as a critical first step to developing spatially explicit canopy and surface fuel maps for silvicultural prescription development and evaluation using next-generation three-dimensional fire behavior and effects models. The identification of individual tree locations and properties needs to be linked with estimates of biomass and other intrinsic fuel properties to develop these maps. Such linkages to crown biomass could be made through existing allometric relationships (Campbell et al., 2023). Such an advance in characterizing the spatial distribution of canopy fuels can also help with surface fuel modeling by linking canopy position with empirical and mechanistic models of surface fuels (McDanold et al., 2023; Sánchez-López et al., 2023). As newer fuel modeling approaches become able to utilize individual tree identification approaches to build three-dimensional representations of the fuels complex for next-generation fire behavior and effects modeling, land managers will be able to increasingly understand and account for spatial heterogeneity while designing and evaluating heterogeneous silvicultural prescriptions.

In addition to providing spatially explicit data for treatment design, and implementation, the data produced by UAS could also inform wildlife habitat management. In the past, LiDAR observations have been proposed for similar purposes in monitoring habitat distributions for species of conservation interest (Vogeler & Cohen, 2016). Across many ponderosa pine-dominated forests, species like the northern goshawk are of particular forest management concern, with forest structure characteristics considered to be a primary limiting factor. Northern goshawks preferentially select centrally located nesting sites in areas with dense patches of old growth and high canopy cover with lifted crowns for sub-canopy flight within the range of the principal prey habitat (Reynolds et al., 1992, 2006). In ponderosa-dominated ecosystems, the principal prey habitat occurs in mosaics of tree clumps of varying maturities and large grass/forb-dominated meadows and interspaces (Reynolds et al., 1992). As a result, habitat management recommendations for the northern goshawk (Reynolds et al., 1992) promote a shifting mosaic of interspersed tree groups in different vegetative structural stages (Reynolds et al., 1992, 2006). However, adequate implementation of these recommendations requires a spatially explicit understanding of horizontal and vertical forest structure. The methods outlined in this study could be reliably scaled, with most consumer-grade UAS capable of capturing 10–20 ha of remote sensing observations in a single flight of 10–20 min (Tinkham et al., 2021). Broader testing of these methods across larger extents could provide the necessary information for guiding treatment implementation landscapes. Should these methods prove able to provide reliable tree-level observations across forest gradients, their potential for augmenting the training data used by satellite-based sensors for mapping forest structure across entire landscapes is immense. Such UAS strategies could provide an order of magnitude increase in the amount of training data available for tuning satellite models compared to traditional ground-based forest inventories.

### Limitations and potential sources of error

As with all inventory strategies, the accuracy of our results needs to be interpreted within the context of their application and the dataset used to validate them. Our use of 1-ha stem maps located within treated stands likely increased the false positive rate within larger tree sizes as the crowns of these trees were observed to overlap into the study area and were extracted as actual trees. However, scaling these methods to full management units and utilizing common stand boundaries like roads should reduce this effect during operational monitoring. Additionally, studies have also found that field observations tend to underestimate tree heights by about 5% and can vary in precision by 10% (Vastaranta et al., 2009). Similarly, Krause et al. (2019) found that field measurements generally misestimated tree heights with a RMSE of 0.30 m and a systematic error of 0.14 m. Such a bias could be a consistent source of error in accuracy assessments and potentially lead to the misinterpretation of results. These types of errors are likely compounded by the 3 years separating the stem map and UAS data acquisitions and contribute to the high overestimation of understory tree density.

The study’s use of ANOVA for comparing the stand-level metrics (e.g., number of clumps, crown area, and distance to a tree) is potentially problematic for only having a sample size of 11. While ANOVA is considered a robust test against deviations from normality when sample sizes are small but equal (Sullivan et al., 2016), it is possible that the small sample size for these metrics may have masked potential significant differences. The Bonferroni adjusted *p*-values for number of clumps and distance to a tree likely indicate the small sample size was not a problem for these metrics. However, the relatively small *p*-values for the crown area comparisons could indicate that there is more departure between the UAS estimates of crown area in each clump class from the field observations than the ANOVA indicates.

## Conclusion

As management objectives in dry conifer forests shift toward promoting horizontal and vertical complexity, there is a growing need for forest inventory techniques capable of capturing the resolution, extent, and spatial explicitness required to inform management decisions. This study found that in relatively open-canopy forests, UAS SfM can successfully detect individual trees from most size classes and estimate tree-level height and DBH across all size classes. This data could be reliably summarized to estimate stand-level density and cover, with the largest errors in the estimation of understory TPH due to issues separating interlocking small tree crowns from each other. Additionally, the data could be summarized to characterize and describe individuals, clumps, and openings as well as inter-clump characteristics like the percent of stand basal area and height CV through all clump size classes. These findings indicate that aerial SfM photogrammetry can effectively characterize large- and small-scale forest structure metrics within ponderosa pine-dominated stands to a level likely adequate for monitoring and implementing spatially explicit management objectives. This approach could also be easily integrated into management processes to inform approaches like the individuals, clumps, and openings method of stand prescription development. However, further work is needed to evaluate if incorporating site-specific drivers of height to DBH relationships can improve DBH modeling and how these techniques will transfer to sites with more complex species compositions.

### Supplementary Information

Below is the link to the electronic supplementary material.

Supplementary file1 (DOCX 19 KB)

## Data Availability

Datasets generated during the current study are available from the corresponding author on reasonable request.
