# Supplementary material for: Characterizing heterogeneous forest structure in ponderosa pine forests via UAS-derived structure from motion
Source: Environ Monit Assess. 2024 May 9;196(6):530. doi: 10.1007/s10661-024-12703-1 (PMC11082040; doi:10.1007/s10661-024-12703-1)
Supplement: Supplementary file 1 — Supplementary file1 (DOCX 19 KB) [file 10661_2024_12703_MOESM1_ESM.docx]

**Table A1.** Metashape setting for UAS structure from motion processing in the Align Photos, Optimized Alignment, and Build Dense Cloud steps. Parameter selection was based on testing conducted by Tinkham and Swayze (2021).

| **Parameter** | **Setting** |
| --- | --- |
| Align Photos |  |
| Accuracy | Highest |
| Generic Preselection | Yes |
| Reference Preselection | Source |
| Reset Current Alignment | No |
| Key Point Limit | 40,000 |
| Tie Point Limit | 4,000 |
| Apply Mask To | None |
| Exclude Stationary Tie Points | Yes |
| Guided Image Matching | No |
| Adaptive Camera Model Fitting | Yes |
| Optimized Alignment |  |
| Fit F | Yes |
| Fit cx, cy | Yes |
| Fit b1, b2 | No |
| Fit K1, K2, K3 | Yes |
| Fit K4 | No |
| Fit p1, p2 | Yes |
| Fit Additional Corrections | No |
| Adaptive Camera Model Fitting | Yes |
| Build Dense Cloud |  |
| Quality | High |
| Reuse depth maps | Yes |
| Depth Filtering | Mild |
| Calculate point colour | Yes |
| Calculate point confidence | No |
